# Design Strategy for Nanostructured Arrays of Metallodielectric Cuboids to Systematically Tune the Optical Response and Eliminate Spurious Bulk Effects in Plasmonic Biosensors

**DOI:** 10.3390/bioengineering9020063

**Published:** 2022-02-04

**Authors:** Anna Luise Grab, Andreas Bacher, Alexander Nesterov-Mueller, Reiner Dahint

**Affiliations:** 1 Applied Physical Chemistry, Institute for Physical Chemistry, Heidelberg University, Im Neuenheimer Feld 253, 69120 Heidelberg, Germany; annaluise.grab@med.uni-heidelberg.de; 2 Clinical Cooperation Unit Molecular Hematology/Oncology, DKFZ Heidelberg and Translational Myeloma Research Group, Department of Internal Medicine V, University Hospital, 69120 Heidelberg, Germany; 3 Karlsruhe Nano Micro Facility (KNMF), Karlsruhe Institute of Technology (KIT), 76344 Eggenstein-Leopoldshafen, Germany; andreas.bacher@kit.edu; 4 Karlsruhe Institute of Technology, Institute of Microstructure Technology, Hermann-von-Helmholtz-Platz 1, 76344 Eggenstein-Leopoldshafen, Germany

**Keywords:** biosensor, optimization, electron beam lithography, surface plasmon resonance, protein adsorption, nanostructure

## Abstract

Plasmonic biosensors are a powerful tool for studying molecule adsorption label-free and with high sensitivity. Here, we present a systematic study on the optical properties of strictly regular nanostructures composed of metallodielectric cuboids with the aim to deliberately tune their optical response and improve their biosensing performance. In addition, the patterns were tested for their potential to eliminate spurious effects from sensor response, caused by refractive index changes in the bulk solution. Shifts in the plasmonic spectrum are exclusively caused by the adsorbing molecules. For this purpose, nanopatterns of interconnected and separated cubes with dimensions ranging from 150 to 600 nm have been fabricated from poly(methyl methacrylate) using electron-beam lithography followed by metallization with gold. It is shown that a small lateral pattern size, a high aspect ratio, and short connection lengths are favorable to generate extinction spectra with well-separated and pronounced peaks. Furthermore, for selected nanostructures, we have been able to identify reflection angles for which the influence of the bulk refractive index on the position of the plasmonic peaks is negligible. It is shown that sensor operation under these angles enables monitoring of in situ biomolecule adsorption with high sensitivity providing a promising tool for high-throughput applications.

## 1. Introduction

In recent decades, plasmonic sensors have been established as a powerful tool for label-free studies of molecular binding processes [1,2,3]. The underlying mechanism is based on the detection of refractive index changes in the sensor environment by resonant coupling of electromagnetic waves to collective oscillations of free electrons in metals leading to localized surface plasmons in metal nanoparticles or propagating surface plasmons in the case of plain metal films [4]. This can be simply achieved by the reflection of light from the sensor surface, yielding specific adsorption bands at wavelengths, where the dispersion curves of plasmon and photon intersect [5,6,7]. To meet the excitation condition, prism or grating couplers [8] have typically been used for propagating plasmonic excitations as well as metal nanostructures for localized surface plasmons [4,9,10]. Refractive index changes in the vicinity of the sensor surface result in wavelength shifts of the plasmonic spectrum. In the case of molecule binding, these effects correlate with the amount of surface-bound species, thus, providing a label-free detection mechanism [11,12].

Among the different types of plasmonic sensors, metal-dielectric nanocomposites have demonstrated high sensitivity down to single-molecule detection, as reported recently [13]. However, when monitoring adsorption or binding processes in situ, shifts in the plasmonic spectrum are not only caused by surface-bound molecules but also by changes in the bulk refractive index of the solutions, which may, e.g., occur upon solution exchange and deteriorate the results. Core-shell nanoparticle-based sensors [14,15,16,17,18] have been shown to overcome this problem for specific angles of light incidence. At a so-called “magic angle”, [19] molecule adsorption is still detected while the device is insensitive to bulk refractive index changes [20,21].

Core-shell nanoparticle-based devices consist of dielectric core particles deposited onto a metal-coated substrate and engulfed with a thin metal shell. They can be prepared by self-assembly processes on a large lateral scale in a time- and cost-effective way [19,21,22]. Upon external reflection of white light and without any need for prisms or grating couplers, they exhibit pronounced extinction spectra with several sharp peaks in the visible regime, which wavelength position can be tuned by appropriate selection of core particle size. The sensitivity of the devices can be optimized by adjusting the shell-to-core ratio and the angle of light reflection. However, the mechanisms behind the formation of the extinction patterns are still not fully understood [23,24,25,26].

In order to modulate the optical response of the self-assembled particles, we used highly regular structures fabricated with electron beam lithography (EBL) [27]. For this purpose, arrays of interconnected and separated dielectric cuboids were fabricated and metalized from the top. In such a way, we achieve strictly regular nanopatterns and overcome a major problem of the self-assembled, less ordered core-shell nanoparticle-based detection system as the optical properties of the layers depend on packing density. Thus, structural inhomogeneities may lead to local changes in optical properties and variations in sensitivity across the device. This, in turn, may cause severe problems in the parallel readout of binding events in high-density arrays, where reproducible optical response all over the sensor surface is required for reliable data interpretation. Especially, the quantification of the amount of surface-bound molecules determined from the observed wavelength shifts needs consistent sensitivity [22].

In addition, the diversity of patterns accessible by EBL is much larger than for self-assembly processes, as not only the height, width, and length of the nanostructured elements but also their separation can be deliberately varied. Moreover, theoretical modeling of the optical properties to provide guidelines for improved sensor design [4,28] is simplified by the regular structure. We, therefore, investigated an alternative to self-assembled core-shell nanoparticle devices based on EBL and fabricated strictly regular, plasmonic nanostructures in the form of cuboids. Lateral size, height, and center-to-center spacing of the cuboids were systematically varied, and extinction spectra of connected (c) and non-connected (nc) cuboids were compared. On this basis, we identify structural parameters, which result in improved optical response and high sensitivity towards molecular adsorption processes. 

## 2. Materials and Methods

### 2.1. Materials

Polystyrene (PS) latex particles with a nominal diameter of 570 nm (standard derivation 10–15%, 10 vol% in water) were purchased from Varian Inc. (Santa Clara, CA, USA). Gold-coated silicon wafers and glass slides for PS particle deposition were obtained from Georg Albert PVD-Beschichtungen (Silz, Germany). They were fabricated by evaporating first a 5 nm layer of titanium as an adhesion promoter and subsequently a 100 nm layer of gold at a pressure of approximately 10^−7^ mbar. Polyethyleneimine (PEI, MW 25.000 Da), phosphate-buffered saline (PBS) tablets, NaCl, TritonX-100, sodium dodecyl sulfate (SDS), and fibrinogen (from human plasma, lyophilized powder, 58% protein) were purchased from Sigma-Aldrich (Taufkirchen, Germany). Poly(methyl methacrylate (PMMA, 99% purity) was ordered from Allresist (Strausberg, Germany). Glycerol was bought from Th. Geyer GmbH (Renningen, Germany). Deionized (DI) water was purified with a Milli-Q plus system from Millipore (Eschborn, Germany) to obtain a specific resistance >18.2 MOhm∙cm. PBS buffer was prepared by dissolving 1 PBS tablet in 200 mL purified DI water.

### 2.2. Fabrication of Nanoarrays by Electron Beam Lithography (EBL)

**Figure 1:**
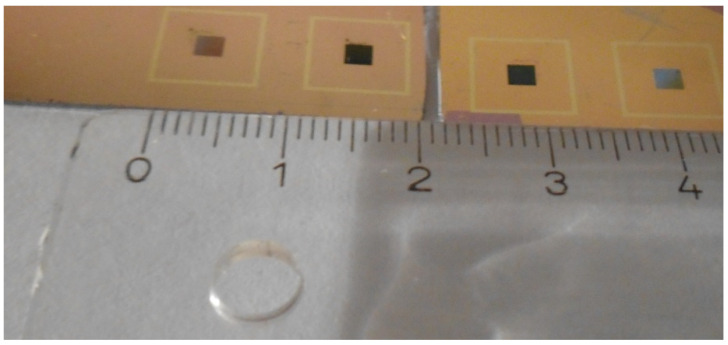
Photo of the silicon wafer after EBL and metallization. The dark squares contain the nanopatterns and are separated from neighboring squares by 1 cm to facilitate cutting the wafer into pieces after fabrication.

Silicon wafers with a diameter of 4 inches and roughened backside were used for spin coating of PMMA and consecutive EBL. The layout for the writing process was chosen in such a way that 4 structures of different geometry were written in 4 repeats, each on the same wafer yielding a 4 × 4 pattern. Each structure had a quadratic shape with an edge length of 2 mm. The distance in between the structures was 1 cm, thus that they could be separated with a glasscutter without damaging them (Figure 1). The writing time per silicon wafer was 102 h.

First, the silicon wafers were coated with 5 nm chromium, serving as an adhesion promotor, and 15 nm gold by vapor deposition at the Karlsruhe Nano Micro Facility (KNMF; Karlsruhe Institute for Technology, Karlsruhe, Germany). Plain PMMA films of 150 nm, 300 nm, and 600 nm thickness were spin-coated on top of the substrates, and the wafers were tempered at 180 °C for 5 min. Next, the nanostructures consisting of interconnected or separated cuboids were generated by mask-less EBL. Afterward, the substrate was tempered for 5 min at 110 °C and developed for 30 s. Post-development time was 15 s. The structures were either left without metallization or evaporated with 5 nm chromium and 30 nm gold. The lateral size and height of the cuboids were varied from 150 to 600 nm, and their separation from 150 to 900 nm.

### 2.3. Fabrication of Core-Shell Nanoparticle Films

PS nanoparticles (PS-NPs) were deposited as hexagonal densely packed monolayers on either gold-coated glass slides or silicon wafers as described previously [29]. In short, the substrate was cleaned with a UV light source (Heraeus Noblelight, Hanau, Germany) and incubated for 20 min with a PEI solution consisting of 2 parts 0.2 mg PEI in 1 mL 1M NaCl solution and 1 part PBS. PS particles were centrifuged, and the supernatant was removed and resuspended in double the amount of ethanol. The particles were transferred to the surface of a water-filled vessel, where they formed a dense monolayer after the addition of SDS and TritonX. In the last step, the film was taken up by the PEI functionalized substrate and metalized with gold nanoparticles (AuNPs) via seeding and plating. [14,15,29] Alternatively, a metal shell (5 nm chromium and 30 nm gold) was deposited on the PS cores with 570 nm diameter via sputter coating (MED 020 Coating System, Bal-Tec AG, Balzers, Liechtenstein) [22].

### 2.4. Protein Adsorption and Bulk Refractive Index Studies

The sensitivity of the nanostructured surfaces towards protein adsorption was investigated with fibrinogen. Fibrinogen is a common model protein for such sensitivity studies due to its biomedical relevance and its strong non-specific interaction with a variety of surface materials. In the majority of the studies, the sensor surface was covered with 1 mg/mL fibrinogen dissolved in PBS for 1 h at room temperature, washed extensively with PBS afterward, and dried in a flow of nitrogen. Extinction spectra were taken before and after protein adsorption with the program SpectraSuite (Ocean Optics, Dunedin, FL, USA), and the peak shift was determined using Origin software (OriginLab Cooperation, Northampton, MA, USA). These measurements in air turned out to be very effective for investigating a large number of samples. For each type of nanostructure, 3 independent measurements were performed using 3 individual sensors of the same geometry.

**Figure 2:**
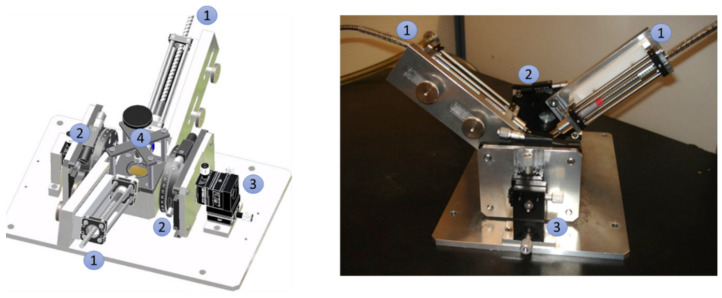
Schematic sketch (**left**) and photo (**right**) of the experimental setup for the adsorption measurements. The optical fibers (1) were mounted on a rotary stage (2) in order to control the incidence angle of light. The fibers were focused onto the sensor surface using an x,y,z-stage (3), and the sensor was placed in a sample holder (4) with a beaker of about 5 cm diameter.

In situ studies on protein adsorption were performed for selected samples and conducted in a glass beaker with the reflection setup shown in Figure 2 and described in Section 2.6. The sensor surface was placed on the bottom of the beaker and exposed to PBS. Next, the optical fibers were immersed into the liquid and adjusted with respect to the sensor surface using a xyz-stage (Thorlabs, Bergkirchen, Germany). After reaching equilibrium, PBS was replaced with a 1 mg/mL solution of fibrinogen in PBS until a steady state was observed. Prior to successive measurements in air, the protein solution was highly diluted and the substrate excessively rinsed with PBS upon removal from solution to avoid Langmuir–Blodgett-like protein deposition at the air-water interface. Finally, the substrate was dried in a flow of nitrogen. The sensitivity towards bulk refractive index changes was investigated with the same setup for different angles of light incidence using glycerol/Milli-Q water mixtures of different volume ratios, resulting in different refractive indices, which were measured with an Abbe refractometer (3T, Atago, Tokyo, Japan).

### 2.5. Scanning Electron Microscopy

The fabricated nanostructures were visualized by scanning electron microscopy (SEM) with a LEO 1530 Gemini (Zeiss, Germany) at room temperature and pressure lower than 10^−5^ mbar. An acceleration voltage of 5 kV was applied, and images were taken using the secondary electron detector (SE2).

### 2.6. Optical Measurements

All optical measurements were performed in reflection geometry (Figure 2) using a combined deuterium tungsten light source (DH-2000 BAL, Ocean Optics, Dunedin, FL, USA) covering a spectral range from 230–2500 nm. Optical fibers (QR600-7-SR125BX, Ocean Optics for normal light incidence, QP400-2-SR-BX and QP600-2-SR-BX, Ocean Optics otherwise) guided the light from the light source to the sample and from the sample to a UV-Vis spectrometer (HR 4000, Ocean Optics), which served as the detector [29].

In brief, the spectra were taken with the program SpectraSuite from Ocean Optics [30]. First, with the light source turned off, a dark spectrum *D*(λ) was measured, followed by an absorption spectrum *R*(λ) of the reference sample, which was a silicon wafer coated with 5 nm chromium and 15 nm gold without nanostructure. Next, the reference sample was replaced with the sensor surface, and its absorption spectrum, *S*(λ), was taken. The extinction spectrum *A*(λ) is then given by

(1) $$A\left(\lambda \right)=-log\left(\frac{S\left(\lambda \right)-D\left(\lambda \right)}{R\left(\lambda \right)-D\left(\lambda \right)}\right).$$

Here, *S*(λ), *D*(λ), and *R*(λ) denote the sample intensity, dark intensity, and reference intensity for a given wavelength λ.

### 2.7. Data Analysis

Maxwell equations are the basis for the description of all light scattering problems. Therefore, several approaches are available, such as effective medium-theory according to Maxwell Garnet [31] or Bruggeman, discrete dipole approximation, boundary element method (BEM) [32], Finite Difference Time Domain Simulation (FDTD) [33], and rigorous diffraction analysis in the Fourier space. The latter is used to calculate selected extinction spectra of cuboids. We used the program DiffractMOD from RSOFT (RSoft Design UK, Baintree, Essex, UK). This program was optimized according to the manufacturer’s protocol for dielectric gratings. The geometry and light incidence angle were defined in a 3D CAD program, and convergence studies were performed as published previously [4].

Briefly, the calculation of the extinction spectrum for periodic structures is conducted in different steps. Step one is the definition of the structure geometry using the 3D CAD- based user interface of the program DiffractMOD. This interface provides the option to choose standard forms from templates (e.g., cubes, squares, circles, or planes). Each layer must be defined individually, including the material parameters. However, due to the periodicity of the grating structure, only the smallest structure size, the so-called elementary cell, needs to be defined in terms of dimensions, thickness, material, and optical parameters. The elementary cell structure has a vertically homogeneous dielectric function, and the periodicity of the structure is reflected in the periodicity of the electrical fields in x-y-direction during the simulation process. For our calculation, we used a refractive index resolution of 0.0045 RIU (RIU = refractive index unit). Lastly, the light wavelength and reflection angle is chosen. We used wavelength steps of 10 nm to calculate the spectrum for light incidence along the surface normal. The structure itself is approximated by steps according to Li et al. [34,35,36], and the DiffractMOD simulations solve the Maxwell equations with periodic boundary conditions based on Coupled Wave Analysis (RCWA) and Modal Transmission Line (MTL) Theory. For details, we refer to RSoft Design Group user manuals [37]. As an output we calculated the extinction spectrum of selected cuboid nanostructures with the same lateral dimensions of 300 nm but different heights (150 nm, 300 nm, 600 nm) and compared it to the optical measurements.

## 3. Results

**Figure 3:**
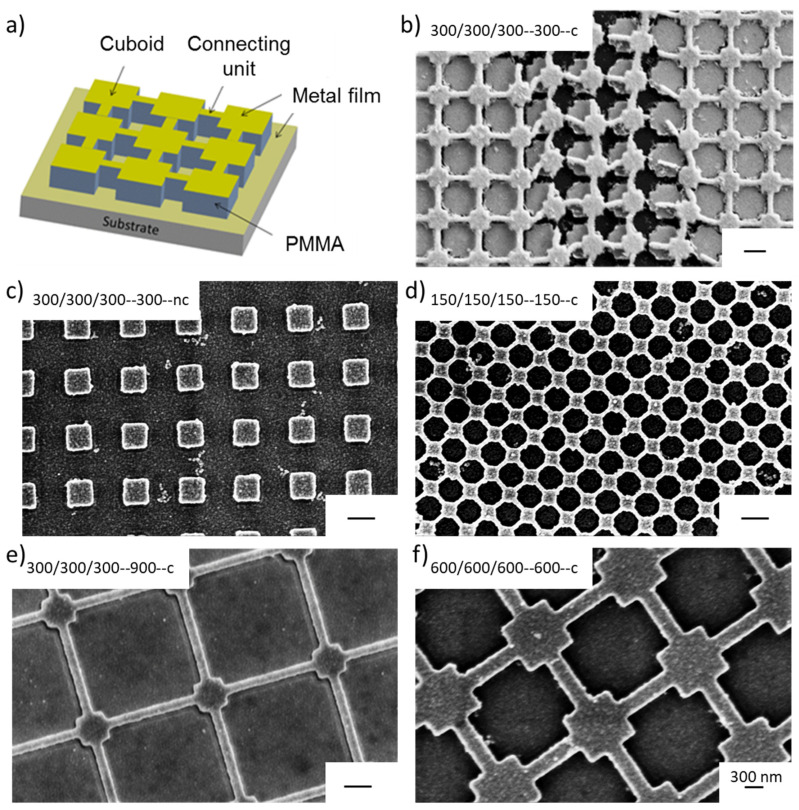
(**a**) General layout of the EBL-fabricated nanostructured arrays. (**b**) SEM image of a 300/300/300-300-c nanoarray, which has been fabricated without chromium as an adhesion promotor after exposure to an aqueous solution. (**c**–**f**) Example REM images of not connected and interconnected nanostructures. The scalebar is 300 nm.

EBL was used to generate strictly regular nanostructures of interconnected and separated cuboids. The general layout is shown in Figure 3a. As substrates, silicon wafers coated with 5 nm chromium and 15 nm Au were used. The use of chromium as an adhesion promotor turned out to be essential in the EBL fabrication process as otherwise, the nanoarrays were destroyed upon liquid contact (Figure 3b). To systematically study the effect of structural dimensions on the corresponding plasmonic spectra and the sensitivity of the arrays towards molecule adsorption, the length (*l*), width (*w*), height (*h*), and separation (*s*) of the cuboids in the nanostructured arrays were varied. The notation *l/w/h-s-x* is used to specify their structural parameters. For connected nanostructures (*x* = *c*), *s* corresponds to the length of the interconnecting units, for not connected nanostructures (*x* = *nc*) to the size of the gap in between the cuboids. All dimensions are given in nm. Example SEM images of different nanoarrays are presented in Figure 3c–f and show the high accuracy of the patterns formed. An overview of the fabricated nanoarrays is given in Table 1.

### 3.1. Comparison of EBL-Generated Nanostructures to Core-Shell Nanoparticle Films and Non-Structured PMMA Layers

**Figure 4:**
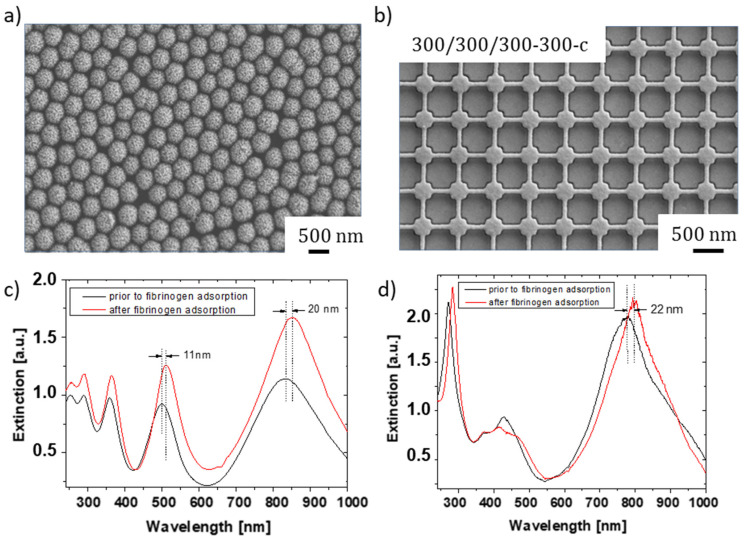
SEM images (top) and redshift of the extinction spectrum upon fibrinogen adsorption (bottom) for (**a**,**c**) core-shell nanoparticle films with a diameter of 570 nm and (**b**,**d**) cuboid nanostructures with an edge length of 300 nm. The plasmonic spectra were measured in air before (black) and after (red) fibrinogen protein adsorption with light incidence along the sensor normal.

In order to evaluate whether EBL-generated plasmonic nanostructures are a promising alternative to core-shell nanoparticle monolayers formed by self-assembly, a metalized 300/300/300-300-c nanoarray was first fabricated for comparative experiments. This array also served as a reference structure for the evaluation of nanoarrays with other dimensions later on. The core-shell nanoparticle film was formed from PS nanoparticles of 570 nm in diameter and metalized with AuNPs via seeding and plating. The corresponding SEM images are shown in Figure 4a,b. Both structures exhibited pronounced extinction peaks in the ultraviolet (UV) and visible (Vis) regime (Figure 4c,d). The optical density of the extinction peaks was even higher for the EBL-generated nanostructure. Protein adsorption onto the nanostructures using a 1 mg/mL solution of fibrinogen in PBS resulted in a redshift of the peaks. With about 20 nm for the core-shell nanoparticle layer and 22 nm for the 300/300/300-300-c nanopattern, the wavelength shift of the peak located at about 800 nm is very similar, indicating that the EBL-generated nanoarrays must be considered as a promising alternative to the core-shell nanoparticle films.

**Figure 5:**
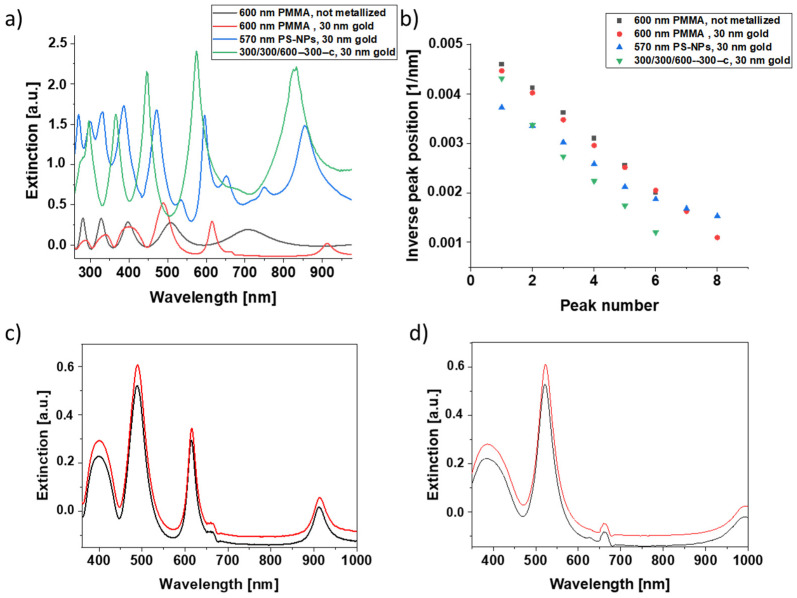
(**a**) Extinction spectra of non-structured and nanostructured PMMA layers and a core-shell nanoparticle film metalized with 30 nm gold by sputter coating. (**b**) Inverse extinction peak position versus peak number for the extinction spectra shown in (**a**). Symbols indicate experimental data. In the case of sole interference, a linear relationship is expected. Optical spectra before (black curve) and after (red curve) protein adsorption onto (**c**) a metallized 600 nm and (**d**) a metallized 300 nm PMMA film. Spectra were detected for light incidence along the sensor normal in air.

In continuation of the comparison, we also investigated the optical response and sensitivity towards fibrinogen adsorption of non-structured PMMA films deposited on gold-coated substrates in order to elucidate the effects of nanopatterning. A film thickness of 600 nm was selected, which is close to the height of the core-shell nanoparticle layers. For non-metalized, plane PMMA films, the optical density is significantly lower than for nanostructured samples (Figure 5a). A plot of the inverse peak position as a function of peak number (Figure 5b) yields a straight line. We may, therefore, conclude that in the case of plain films, the observed extinction spectrum is dominated by Bragg interference. For normal light incidence, the Bragg equation reduces to 2∙*d* = *p*∙*λ*/*n*, where *d* denotes the layer thickness, *p* the peak number, and *n* the refractive index of the layer, so that 1/*λ* = *p*/(2∙*d*∙*n*). From the slope of the curve in Figure 3b, where 1/*λ* is plotted over *p*, we, thus, obtain *d* ≈ 650 nm if we assume a refractive index of 1.49 for the PMMA layer [38]. The calculated thickness value is, therefore, in good agreement with the nominal PMMA thickness of 600 nm, confirming the above conclusion.

Metallization of the PMMA layer leads to more pronounced variations in peak height and a peak shift, especially for higher peak numbers (Figure 3a). This increase in peak intensity improves the general structure of the extinction spectrum and could be a consequence of enhanced interference effects. Strikingly, the height of the peaks is still significantly lower than for core-shell nanoparticle layers. Furthermore, plane PMMA films only showed minor peaks shifts in response to fibrinogen adsorption (Figure 5c,d). Thus, nanostructured films (EBL-generated or core-shell nanoparticle-based) are clearly advantageous for sensing applications. As already observed and discussed for core-shell nanoparticle sensors [22], a plot of inverse extinction peak position versus peak number shows an almost linear behavior (Figure 5b), suggesting the contribution of interference effects to the optical response also for EBL-generated nanostructures.

### 3.2. Design Strategies for EBL-Generated Plasmonic Nanostructures

Having shown that EBL-generated nanoarrays are a promising alternative to self-assembled core-shell nanoparticle sensors, efforts were made to optimize their plasmonic response by variation of their lateral dimensions. For this purpose, the higher flexibility in pattern generation compared to core-shell nanoparticle films was exploited.

**Figure 6:**
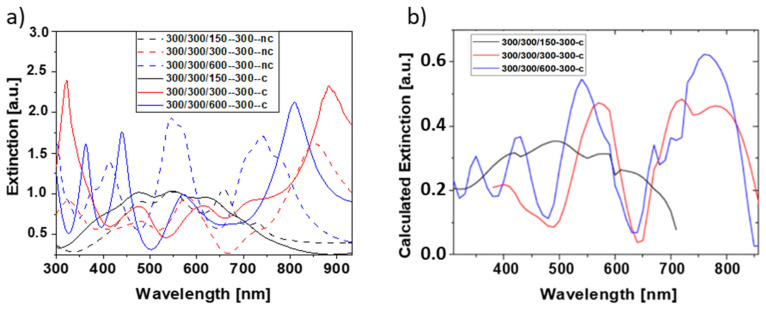
Extinction spectra of cuboid-shaped nanostructures with the same lateral dimensions of 300 nm but different height (150 nm black, 300 nm red, 600 nm blue). (**a**) Measured for nanostructures with (line) and without (dashed) interconnections and (**b**) calculated for the cuboid nanostructures with interconnections. Spectra were detected for light incidence along the sensor normal in air.

First, we investigated the influence of structure height and cube interconnections on the extinction properties. For this purpose, the extinction spectra of cuboids with 300 nm width and length but different heights (150 nm, 300 nm, 600 nm) were measured for light incidence parallel to the surface normal in the air (Figure 6a). Several resonances were observed whose position and shape depended on the structure height. While for a structure height of 150 nm, broad peaks with comparatively low extinction values were found, pronounced peaks were observed for higher thickness values. This observation holds for both interconnected and not connected nanostructures. Overall, high thickness values seem to be favorable in order to obtain extinction spectra with a number of pronounced peaks distributed all over the investigated wavelength regime. This finding agrees with numerical calculations (Figure 6b) based on rigorous diffraction analysis (RCWA) using RSOFT.

**Figure 7:**
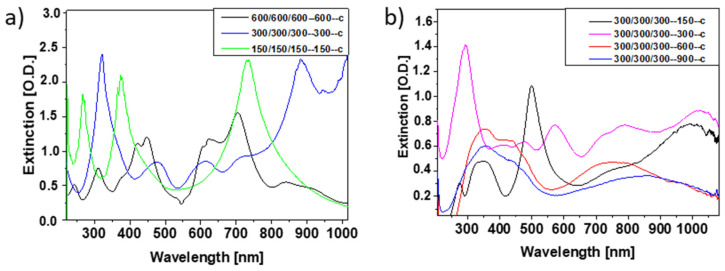
Extinction spectra of (**a**) different cube-shaped nanostructures scaled in size and (**b**) cube-shaped nanostructures with different lengths of interconnecting units. All nanostructures are interconnected. Spectra were detected for light incidence along the sensor normal in air.

Scaling of the 300/300/300-300-c reference structure by a factor of 0.5 and 2, i.e., the formation of cubes with dimensions of 150 and 600 nm, respectively, clearly shows the most pronounced extinction spectrum for the smallest pattern size (Figure 7a). The optical properties can also be improved upon changing from cubes to cuboids whose height is larger than their width and length (Figure 6) and for small cuboid separation (Figure 7b). We may, therefore, conclude that small pattern size, a high aspect ratio, and short connection lengths result in well-separated pronounced peaks.

### 3.3. Sensitivity to Bulk Refractive Index Changes

Another important characteristic of the above core-shell nanoparticle layers is the existence of a so-called “magic angle” of light incidence for which the extinction spectrum is insensitive to refractive index changes of the adjacent bulk solution while molecule adsorption can still be monitored [22]. In such a way, spurious peak shifts related to changes in solution composition can be clearly separated from those caused by binding processes, which is an important aspect of sensor development. Thus, the question arises of whether the EBL-generated nanostructures exhibit the same feature.

To address this question, arrays of interconnected and not connected cuboids were exposed to water/glycerol mixtures of the different bulk refractive index under different light reflection angles. Cuboid length and width were kept constant at 150, 300 or 600 nm, while their height ranged from 150 to 600 nm. The refractive index, *n*, of the solutions was varied from 1.33 to 1.43 RIU by mixing glycerol and water in different ratios. From the wavelength shift, Δ*λ*, of the plasmonic peak at 600 nm as a function of solution refractive index change, Δ*n*, the bulk sensitivity *S* = Δ*λ*/Δ*n* of the nanoarrays was determined for various angles of light incidence [22,29]. The magic angle is reached when the bulk sensitivity is zero. For this angle, the position of the extinction peak is not affected by any changes in the bulk refractive index.

**Figure 8:**
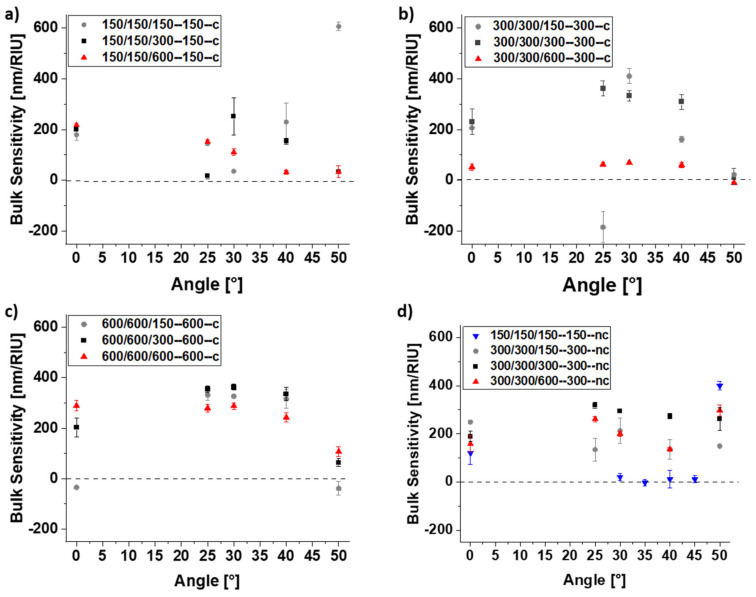
Sensitivity of EBL-generated nanostructures towards refractive index changes of the bulk solution for interconnected nanostructures with lateral dimensions of (**a**) 150 nm, (**b**) 300 nm, and (**c**) 600 nm and different heights (150 nm, 300 nm, and 600 nm). The connection length corresponds to the respective lateral dimensions. (**d**) Sensitivity values for several non-connected nanostructures. For some structures, a so-called “magic angle” exists for which the system is insensitive to (spurious) bulk refractive index changes.

As shown in Figure 8a–d for arrays of interconnected and non-connected cuboids, such a magic angle is only observed for selected geometries. Interestingly, interconnections may be of significant importance for its existence. As seen from Figure 8b,d (structures 300/300/300-300-c and 300-300-300-nc, respectively), the magic angle may vanish if the connecting parts of the nanostructure are removed. Interconnected structures with an edge length and separation of 150 nm (150/150/150-150-c) exhibit sharp extinction peaks (Figure 7a) and a maximum bulk sensitivity of 200 nm/RIU (Figure 8a). For these structures, the magic angle is not reached, but bulk effects are negligible for light incidence at 30°. Low bulk sensitivity values are also observed at 25° for the same type of sensor with height increased by a factor of 2 (150/150/300-150-c) and at 40° for height increased by a factor of 4 (150/150/600-150-c) (Figure 8a). For interconnected structures with edge length and separation of 300 nm (Figure 8b), the magic angle is 50° and independent of the structure height. 50° is also the magic angle for structure 600/600/150-600-c, while for higher structures of the same type, the bulk sensitivity at 50° is still below 100 nm/RIU (Figure 8c).

**Figure 9:**
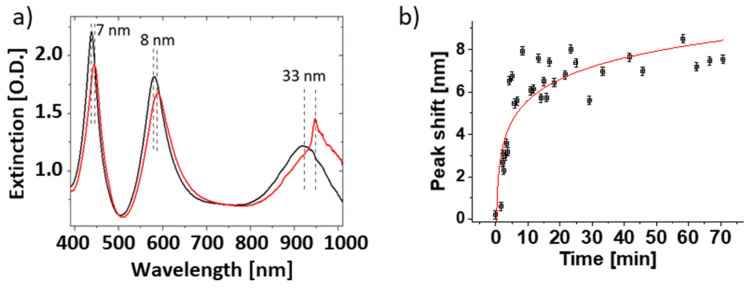
(**a**) Extinction spectra of a 150/150/300-150-c nanostructure prior to (black) and after (red) fibrinogen adsorption taken under normal light incidence in PBS. (**b**) The shift of the peak position of the extinction spectrum over time monitored under the magic angle in PBS.

Finally, various EBL-fabricated nanoarrays were tested for their sensitivity in in situ biosensing applications under liquid (Figure 9a,b). For this purpose, the wavelength shift upon fibrinogen adsorption was directly measured in PBS. In the experiments, we mounted the nanostructures in an open liquid cell, which allowed the exchange of liquid via syringes. After exposing the nanostructures to PBS, the buffer solution was replaced by a 1 mg/mL fibrinogen solution in PBS. After about 1 h incubation time, the protein solution was again exchanged with PBS. Extinction spectra under liquid were taken in PBS for normal light incidence prior to and after protein adsorption. Among the investigated samples, the highest wavelength shifts were obtained for a 150/150/300-300-c nanostructure with values of 7 nm, 8 nm, and 33 nm for the extinction peaks located at about 450, 600, and 950 nm (Figure 9a). These values are about a factor of 2 higher than the wavelength shifts observed for core-shell nanoparticle sensors under similar conditions.

To monitor protein adsorption processes, time-resolved measurements have been performed for the same type of sample. To exclude bulk refractive index changes from the response, light incidence occurred under the magic angle. The peak shift at about 680 nm was monitored as a function of time. As shown in Figure 9b, the adsorption process takes about 1 h and yields a final wavelength shift of about 8 nm. This value is more than three times higher than for our standard core-shell nanoparticle sensors under comparable conditions [21]. The fact that the wavelength shift under the magic angle is smaller than for normal light incidence is consistent with previous observations for core-shell nanoparticle sensors.

## 4. Discussion

Our studies show that EBL-generated nanostructures are promising plasmonic devices, which can be fabricated with excellent lateral homogeneity and reproducibility. Due to the high flexibility in nanopattern formation, their optical properties can be deliberately tuned. With respect to optical response, small lateral pattern size, a high aspect ratio, and short connection lengths appear to be favorable in order to obtain extinction spectra with well-separated and pronounced peaks. In comparison to core-shell nanoparticle-based devices, which are prepared by simple and cost-effective self-assembly on a large lateral scale, but limited in their structural variety, EBL-generated nanostructures are more demanding and costlier in fabrication but provide the opportunity to individually shape their optical response by deliberate variation of the size, shape, and distance of their nanoscale components.

In biosensing applications, EBL-generated nanostructures allow for the in situ monitoring of biomolecule binding. For selected nanopattern geometries, magic angles were found, and fibrinogen adsorption was monitored under these angles, thereby eliminating spurious bulk responses. This phenomenon has originally been reported by Takei et al. for cap-shaped nanoparticle monolayers [19] and also turned out to be a general feature of the core-shell nanoparticle sensors developed by our group [21]. As demonstrated by Takei et al. [19] in both experimental studies and optical simulations, the position of the absorption spectra depended on both the angle of light incidence and the bulk refractive index of the environment. They also showed that a specific angle exists, where the spectra for different refractive indices overlap, while molecule adsorption still results in a shift of the plasmonic peak. We assume that the same mechanisms apply to our EBL-generated nanostructures.

In in situ experiments, the sensitivity of EBL-generated nanostructures towards biomolecule adsorption has been more than three times higher than for our standard core-shell nanoparticle layers [21]. The studies also reveal that nanostructures with pronounced extinction spectra do not necessarily exhibit large wavelength shifts upon bulk refractive index changes or biomolecule adsorption. On the other hand, strong and sharp extinction bands reduce the error in the determination of peak position, thus that the choice of the best-suited sensing element will have to consider both aspects.

Especially for parallel readout of binding events in high-density arrays by plasmonic imaging [39,40], the generation of strictly regular nanopatterns with minimal response to spurious bulk effects is advantageous as reproducible optical response all over the sensor surface is required for reliable data interpretation. To reduce the effort and costs for the production of the devices, replication from a master structure [41] will probably be the most effective approach, e.g., by molding procedures [42]. The option of signal control [4,38,43,44] and biofunctionalization make plasmonic platforms a powerful tool in the field of clinical studies [43,45].

## 5. Conclusions

This work presents for the first time a number of experimental results comparing the optical resonant properties of periodic metallic structures with and without interconnections. The possibility of fabricating such nanoarrays by electron beam lithography was demonstrated. Their optical resonant properties were compared with those for periodic structures formed by nanoparticles self-assembled on surfaces. The EBL-generated nanopatterns have a high potential for the development of label-free biosensors with improved sensitivity and tailored optical response. In the case of an optimal gold nanostructure with interconnections that was used for the detection of fibrinogen, the highest wavelength shift of 33 nm for the extinction peak located at 950 nm was demonstrated. The excellent reproducibility and lateral homogeneity of the nanostructures generated by EBL help to avoid local changes in optical properties and hence variations in sensitivity across the device.

## Figures and Tables

**Table 1:** Dimensions of the nanostructured arrays fabricated by EBL. Note that for structures no. 6–8 and 18–20 connection width differs from 100 nm as these arrays were fabricated by scaling structure no. 14 by a factor of 0.5 and 2, respectively.

Structure	Cube Length	Cube Width	Cube Height	Connections	Gap in between Cuboids		Abbreviation
1	150 nm	150 nm	150 nm	no	150 nm		150/150/150-150-nc
2	150 nm	150 nm	300 nm	no	150 nm		150/150/300-150-nc
3	300 nm	300 nm	150 nm	no	300 nm		300/300/150-300-nc
4	300 nm	300 nm	300 nm	no	300 nm		300/300/300-300-nc
5	300 nm	300 nm	600 nm	no	300 nm		300/300/600-300-nc
Structure	Cube Length	Cube Width	Cube Height	Connection Length	Connection Width	Connection Height	
6	150 nm	150 nm	150 nm	150 nm	50 nm	150 nm	150/150/150-150-c
7	150 nm	150 nm	300 nm	150 nm	50 nm	300 nm	150/150/300-150-c
8	150 nm	150 nm	600 nm	150 nm	50 nm	600 nm	150/150/600-150-c
9	300 nm	300 nm	150 nm	150 nm	100 nm	150 nm	300/300/150-150-c
10	300 nm	300 nm	150 nm	300 nm	100 nm	150 nm	300/300/150-300-c
11	300 nm	300 nm	150 nm	600 nm	100 nm	150 nm	300/300/150-600-c
12	300 nm	300 nm	150 nm	900 nm	100 nm	150 nm	300/300/150-900-c
13	300 nm	300 nm	300 nm	150 nm	100 nm	300 nm	300/300/300-150-c
[14]	300 nm	300 nm	300 nm	300 nm	100 nm	300 nm	300/300/300-300-c
15	300 nm	300 nm	300 nm	600 nm	100 nm	300 nm	300/300/300-600-c
16	300 nm	300 nm	300 nm	900 nm	100 nm	300 nm	300/300/300-900-c
17	300 nm	300 nm	600 nm	300 nm	100 nm	600 nm	300/300/600-300-c
18	600 nm	600 nm	150 nm	600 nm	200 nm	150 nm	600/600/150-600-c
19	600 nm	600 nm	300 nm	600 nm	200 nm	300 nm	600/600/300-600-c
20	600 nm	600 nm	600 nm	600 nm	200 nm	600 nm	600/600/600-600-c

## Data Availability

The data presented in this study are available in data are provided in the article and can additionally be provided upon request.
